# Mr. Bell, on Burns and Scalds

**Published:** 1800-03

**Authors:** 


### *To the Editors of the Medical and Phyſical Journal.*

GENTLEMEN,

I Am glad to obſerve in the Medical and Phyſical Journal, No. XII. p. 191, that a ſubject ſo highly intereſting to every individual as that of burns and ſcalds, continues to draw its due ſhare of attention from the medical world. Mr. Kentiſh,* of Newcaſtle, was, I believe, the firſt who had the merit of bringing the ſubject into notice. As his method of treatment is exactly the reverſe of that of Mr. Earle, I hope you will permit me to occupy a ſmall portion of your valuable work in attempting to explain my reaſons for preferring the practice of the former gentleman to that of the latter.

In very extenſive burns, where nearly the whole ſurface of the body is affected, the patient is always ſeized, ſoon after the accident,

---

* See an Eſſay on Burns, &c. by Edward Kentiſh, Surgeon, London, Robinſons, 1797.

accident, with violent ſhivering, and at the ſame time complains of moſt excruciating burning pain. During this ſtage, the pulſe is ſo ſmall as to be ſcarcely perceptible; and, in many caſes that I have ſeen, it could not be felt at the wriſt. Would it be prudent, whilſt this paroxyſm continues, to plunge the patient into a cold bath, or to apply ice to the whole ſurface of the body? It may be ſaid, that this torpor continues only for a ſhort period. — In reply I muſt obſerve, that I have ſeen a patient with this cold fit upon him for upwards of three hours, and have found it neceſſary to cover him up as warm as poſſible, to give him mulled wine, and to put bottles filled with warm water to his feet.

The ſuperiority of the ſtimulating practice is manifeſted in this, — that when the eſſential oil of turpentine is applied to a ſcalded or burned part, relief is, in moſt caſes that I have ſeen, produced within half an hour, provided that the remedy is made uſe of as ſoon after the accident as poſſible; nor have I obſerved any caſe, under the above circumſtances, where the pain was protracted more than two hours.

In ſeveral ſlight caſes, where I have ſeen cold water made uſe of, it always required ſix, and not unfrequently eight hours to free the ſufferer from agony; for the moment the application of cold water ceaſed, the pain returned with much greater vehemence. I recollect a caſe, which an eminent ſurgeon in Newcaſtle, Mr. Anderſon, communicated to Mr. Kentiſh more than two years ago, and which is moſt decidedly in favour of what is here advanced. A lady had both her arms ſeverely ſcalded with boiling water, from above the elbows down to the fingers' ends. The *ol. terebinth.* was applied to one arm ſoon after the accident, and the other plunged into cold water, which was renewed as often as it became warm. That arm to which the *ol. terebinth.* was applied, became perfectly eaſy in about half an hour; the other continued to give pain when taken out of the water, even for an inſtant, for more than ſix hours; and, as far as I recollect, it required a much longer time for its cure than the other.

[ *For want of room, we ſhall conclude this Letter in our next.* ]

